# State-of-the-Art and Future Challenges for Nutritional Interventions in Facioscapulohumeral Dystrophy: A Narrative Review

**DOI:** 10.3390/nu17061056

**Published:** 2025-03-17

**Authors:** Venere Quintiero, Oscar Crisafulli, Daniele Diotti, Rossella Tupler, Massimo Negro, Emanuela Lavaselli, Giuseppe D’Antona

**Affiliations:** 1 Centro di Ricerca Interdipartimentale nelle Attività Motorie e Sportive (CRIAMS) Sport Medicine Centre Voghera, University of Pavia, 27058 Voghera, Italydaniele.diotti@unipv.it (D.D.);; 2 Department of Life Sciences, University of Modena and Reggio Emilia, 41121 Modena, Italy; 3 Department of Public Health, Experimental and Forensic Medicine, University of Pavia, 27100 Pavia, Italy

**Keywords:** FSHD, muscle atrophy, nutrition, supplements, proteins, leucine, creatine, antioxidants, bioactive compounds

## Abstract

Facioscapulohumeral dystrophy (FSHD), the second most common inherited muscular dystrophy in adulthood, is characterized by progressive muscle loss, accompanied by an increase in fat mass. Beyond these alterations in body composition, which contribute to the risk of sarcopenic obesity, FSHD is associated with systemic inflammation and oxidative stress. These interconnected mechanisms may worsen muscle atrophy, leading to a decline in physical efficiency and quality of life. While the therapeutic benefits of physical therapy and exercise have been investigated, the impact of dietary interventions remains underexplored. Given the established role of nutrition in managing various chronic diseases, there is growing interest in understanding how it might influence the clinical management of FSHD. By addressing current gaps in the literature, this review aims to investigate the potential role of dietary patterns and specific nutrients in modulating muscle metabolism within the context of FSHD. Some studies have indicated various compounds (flavonoids, curcumin, L-carnitine, coenzyme Q10, and omega-3), vitamins (C and E), and minerals (zinc and selenium) with antioxidant and anti-inflammatory properties as promising treatment strategies for FSHD. Instead, few data regarding the effects of proteins and creatine supplementation are available. Furthermore, the potential benefits of essentials amino acids, β-hydroxy-β-methylbutyrate, and vitamin D in contrasting muscle atrophy and enhancing muscle function remain unexplored. Despite these preliminary findings, the existing body of evidence is limited. Identifying novel therapeutic strategies to complement existing treatments could provide a more comprehensive management framework, aimed at improving the long-term health outcomes and quality of life of FSHD patients.

## 1. Introduction

Facioscapulohumeral muscular dystrophy (FSHD) is among the most common hereditary muscular dystrophies, with an estimated prevalence of approximately 1 in 15,000–20,000 individuals [1]. The pathogenesis of FSHD is driven by the aberrant expression of the DUX4 gene, which triggers dysregulation of several downstream pathways, leading to muscle atrophy, increased cellular sensitivity to oxidative stress, and systemic inflammation [2]. Clinically, FSHD is characterized by a progressive loss of muscle mass, accompanied by an increase in fat mass [3]. These alterations in body composition may contribute to functional impairments, including marked fatigue [4], altered gait [5,6], reduced mobility [7], loss of strength [8], and exercise intolerance [9,10,11], as well as an increased risk of sarcopenic obesity [3]. In addition to muscle atrophy, FSHD patients exhibit changes in body fluid distribution, including reduced intracellular water (ICW) and an altered ICW/extracellular water (ECW) ratio [12]. Fluid balance is crucial for muscle health and function [13], and its disruption may worsen muscle atrophy, contributing to the clinical manifestations of the disease [13]. Indeed, alterations in ICW, ICW/ECW, and ECW/ICW ratios have been associated with impaired muscle mass, strength and functionality, hallmark features of FSHD, across various cohorts of patients [14,15,16,17]. Currently, there is no cure for FSHD, and treatment primarily relies on non-pharmacological interventions aimed at alleviating symptoms and contrasting disease progression [18,19]. Such interventions include the use of orthotic devices, which have been associated with a reduced risk of falling [20], neuromuscular electrical stimulation [21], and vibratory therapy [22], which have been associated with improvements in daily activities capacity. Furthermore, several studies have investigated the effects of aerobic [23,24,25] and resistance training [26,27], reporting some improvements in terms of aerobic fitness [23,24], muscle endurance [25], strength [26], and quality of life [27]. However, evidence supporting the benefits of dietary interventions, either alone or combined with exercise, in FSHD patients remains scarce. Furthermore, data on the efficacy of dietary supplements are limited.However, a comprehensive synthesis of the existing evidence would provide a valuable and easily accessible reference to assist clinicians in evaluating the evidence-based efficacy of nutritional interventions, a tool not yet available in the literature. Moreover, identifying gaps in current knowledge could guide future research directions, ultimately opening new avenues for improving the clinical management of FSHD patients. Consequently, this review aims to summarize the available data on nutritional and supplemental interventions in FSHD, address existing knowledge gaps, and propose a rationale for future research priorities.

## 2. Materials and Methods

In this review, we focused on studies exploring nutritional and supplemental strategies for managing FSHD. To identify relevant articles, we searched the PubMed and Web of Science databases using the following search terms: “FSHD”, “facioscapulohumeral muscular dystrophy”, “muscle atrophy”, “inflammation”, “oxidative stress”, “diet”, “nutrition”, “supplements”, “nutraceuticals”, “antioxidants”, “proteins”, “essential amino acids”, “creatine”, and “microbiota”. Observational and intervention studies published in English and addressing the nutritional aspects of FSHD were included. Opinion articles, letters, conference abstracts, and non-peer-reviewed articles were excluded.

## 3. Pathophysiology of FSHD

As previously mentioned, FSHD is caused by the abnormal expression of the DUX4 gene, which is located within the 3.3 kb repeats unit (RUs) of the D4Z4 macrosatellite on chromosome 4q35 [28]. In healthy individuals, the D4Z4 macrosatellite typically consists of 11–100 RUs and is hypermethylated, effectively silencing DUX4 expression [29]. In contrast, in patients with FSHD, the array is contracted to 10 or fewer RUs, and the presence of a permissive 4qA haplotype facilitates the production of a polyadenylated DUX4 transcript [29]. This configuration prevents proper methylation, resulting in the hypomethylation of DUX4 and its pathological activation [29]. As a result, DUX4 triggers a cascade of genetic alterations that disrupt the balance between skeletal muscle protein synthesis (MPS) and muscle protein breakdown (MPB), resulting in the latter exceeding the former, and, consequently, in muscle atrophy [30]. The main mechanisms by which DUX4 induces muscle atrophy include inhibition of myogenic differentiation factor (MyoD), which impairs myoblasts differentiation into mature muscle fibers [31,32], activation of p53-mediated apoptotic pathways, resulting in muscle fiber death [33,34], and disruption of the regenerative capacity of satellite cells, which are crucial for muscle repair [35]. Furthermore, the aberrant expression of the DUX4 gene leads to a range of pathological outcomes, including inflammation, oxidative stress, and mitochondrial dysfunction [36,37], which exacerbate muscle atrophy and create a vicious cycle that accelerates disease progression [2,32].

Muscle atrophy and reduced muscle function may also be aggravated by altered body fluid distribution in FSHD patients [12]. Proper levels of ICW and ECW are crucial for maintaining normal muscle function [13]. ICW is considered an indicator of muscle mass [38], as it represents a significant portion of muscle mass that helps to maintain cellular homeostasis, protein synthesis, and metabolic function [13]. In contrast, ECW, mainly found in the interstitial spaces and blood, plays a key role in nutrient transport and waste removal [13]. The equilibrium between these two compartments is tightly regulated and is crucial for muscle health [13]. A loss of this balance, as observed in FSHD patients [12] and in other cohorts of patients [14,15,16,17], may lead to reduced muscle mass, strength, functionality, and generalized muscle weakness [13]. Specifically, the study by Crisafulli et al. [12] showed that FSHD patients exhibited reduced ICW and a reduced ICW/ECW ratio compared with controls, likely due to muscular impairments and compromised muscle cell membrane integrity. On the other hand, ECW may be relatively increased in patients with FSHD due to inflammation, which increases capillary permeability and leads to the expansion of interstitial space and an increase in the extracellular volume [39].

In addition to the well-known factors influencing muscle protein balance, such as nutrition and exercise [30,40], gut microbiota have been shown to affect pathways involved in protein homeostasis through the production and release of bioactive compounds. Gut microbiota produce metabolites, including short-chain fatty acids (SCFAs), branched-chain amino acids, and bile acids [41], which can exert effects on distal organs, such as muscle [42], influencing both its physiology and pathophysiology [43]. The close interaction between the microbiota and muscle and the fact that alterations in the former (i.e., muscle atrophy) coincide with alterations in the latter (i.e., dysbiosis), and vice versa, have led to the development of the gut–muscle axis hypothesis and the search for strategies that regulate muscle function by modulating the composition of the intestinal microbiota [42]. Based on these considerations, it is plausible to hypothesize that the peculiar muscle atrophy observed in FSHD patients may influence their gut microbiota, and that potential alterations in the microbiota could, in turn, exacerbate the muscle atrophy.

## 4. Available Studies Involving Nutritional Interventions in FSHD

Currently, there is a limited number of studies on nutritional interventions in the FSHD population. Specifically, we identified three intervention studies involving antioxidant and anti-inflammatory supplements [44,45,46], one study on post-exercise protein–carbohydrate supplementation [47], and one on creatine monohydrate supplementation [48]. A summary of the available evidence is presented in Table 1.

### 4.1. Antioxidants and Anti-Inflammatory Supplementation

In a study by Passerieux et al. [44], 53 patients with FSHD were randomly allocated to receive a supplementation of either vitamin C, vitamin E, zinc gluconate, and selenomethionine (micronutrients typically deficient in these patients) or a matching placebo. The supplemented group consisted of 26 patients (15 males and 11 females, with a mean age of 42.4 ± 10.2 years), while the placebo group included 27 patients (13 males and 14 females, with a mean age of 37.6 ± 9.3 years). After 17 weeks of intervention, the supplemented group showed a greater reduction in oxidative stress markers compared with controls. This reduction was associated with improvements in muscle function, specifically in maximal voluntary contraction and quadriceps resistance.

In the study conducted by Sitzia et al. [45], 29 patients with various forms of muscular dystrophies, including five with FSHD (five males, with a mean age of 36.8 ± 9.8 years), were randomly assigned to receive either 24 weeks of supplementation with flavonoids and omega-3 fatty acids (known for their anti-inflammatory and antioxidant properties) as part of the FLAVOMEGA complex, or a matching placebo. The supplementation, which included docosahexaenoic acid (DHA), eicosapentaenoic acid (EPA), vitamin E, lemon essential oil, curcumin, acetyl L-carnitine, ascorbic acid, coenzyme Q10, and green tea catechins, was associated with improvements in the 6 min walk test and knee extension measurements.

Finally, Wilson et al. [46] recently reported that, in a cohort of 20 FSHD patients (mean age 39.1 ± 11.6 years), maintaining the same antioxidant regimen proposed by Passerieux et al. [44] for 17 weeks resulted in enhanced muscle function and composition, including increased strength and muscle mass, and reduced fat volume.

### 4.2. Post-Exercise Protein–Carbohydrate Supplementation

Only one study [47] has investigated the effect of protein–carbohydrate supplementation after aerobic training sessions in patients with FSHD. This study involved 41 patients (21 males and 20 females, with a range age of 19–65 years) who were randomly assigned to one of three groups: a training group with protein–carbohydrate supplementation (*n* = 18), a training group without supplementation (*n* = 13), or a nonintervention control group (*n* = 10). While results showed that 12 weeks of supplementation, assumed within three hours post-session, led to a reduced rate of MPB, no additional improvements in muscle function were observed.

### 4.3. Creatine Monohydrate Supplementation

In a randomized controlled trial by Walter et al. [48], creatine monohydrate supplementation (5 g/day for children and 10 g/day for adults for 8 weeks) was compared with a placebo in patients with various muscular dystrophies, including 12 patients with FSHD (mean age 37 ± 19 years). Creatine supplementation was well tolerated by all participants. Although the overall results showed that creatine treatment was associated with improvements in strength, the benefits observed in the FSHD group were not significant.

## 5. Promising Dietary Pattern and Supplements for Future Research in FSHD

Due to the limited number of studies exploring nutritional interventions in FSHD, this field presents opportunities for new research. In the following sections, we discuss theoretical dietary patterns and supplements targeting some of the clinical features of FSHD, which may serve as promising candidates for future studies in this cohort of patients. Our considerations are based on evidence from populations that share pathological features with FSHD, such as muscle atrophy, inflammation, and oxidative stress. These include other forms of muscular dystrophies (e.g., Duchenne muscular dystrophy) or individuals with sarcopenia. It is important to note that such considerations may not be fully applicable to FSHD; therefore, they should be regarded as suggestive of potential future research directions, rather than being directly applicable to FSHD patients.

### 5.1. The Mediterranean Diet: A Promising Anti-Inflammatory and Antioxidant Approach for FSHD

As previously discussed, FSHD is characterized by inflammation and oxidative stress [12]. While these conditions are not the sole drivers of the disease, they are strongly influenced by non-pharmacological factors, such as diet [49]. The Mediterranean diet, by prioritizing the consumption of fruits, vegetables, whole grains, nuts, legumes, extra virgin olive oil, and fish, provides a rich supply of dietary fiber, vitamins (e.g., β-carotene, vitamin C, vitamin E), minerals (e.g., selenium), bioactive compounds (e.g., polyphenols), and monounsaturated and omega-3 polyunsaturated fatty acids. All together, these dietary components may exert synergistic anti-inflammatory and antioxidant effects [50,51]. Additionally, dietary fiber serves as nourishment for the gut microbiota [52], inducing changes in gut composition, that favor the production of SCFAs with the potential to suppress inflammatory patterns [51]. Due to these properties, the Mediterranean diet has shown beneficial effects in several conditions where inflammation and oxidative stress are prominent [53,54,55]. Moreover, it has been proposed as a dietary model for managing some forms of muscular dystrophies, such as Duchenne muscular dystrophy, where oxidative stress and inflammation play pivotal roles [56]. In light of these considerations, FSHD patients, who typically experience inflammation and oxidative stress, could also potentially benefit from such a dietary pattern. This hypothesis could be an interesting objective for future research.

**Figure 1:**
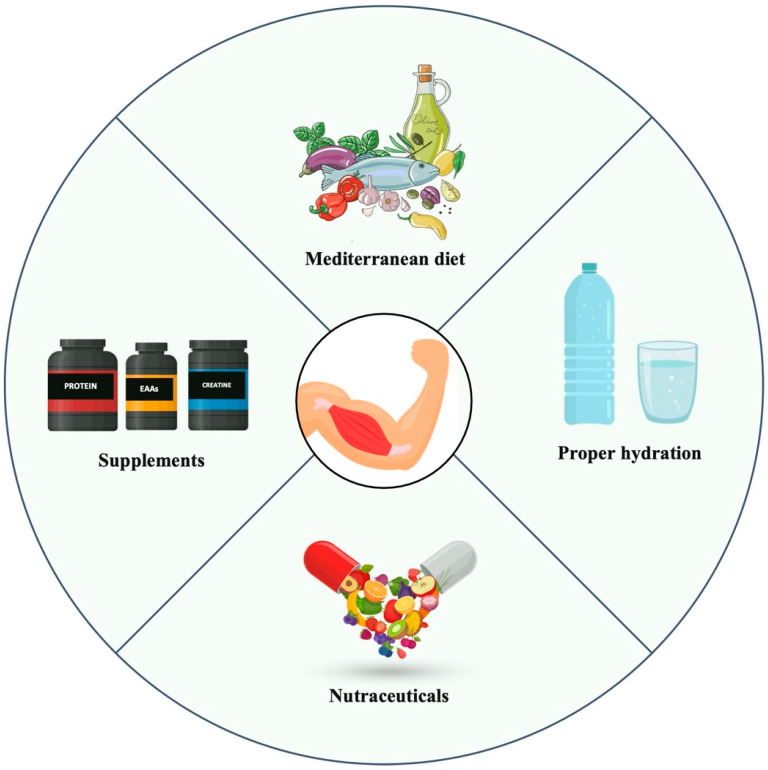
Potential nutritional strategies for muscle health in patients with facioscapulohumeral dystrophy. EAAs, essential amino acids.

In general, a varied and balanced diet, such as the Mediterranean diet, provides sufficient nutrients to meet bodies’ requirements. However, as suggested by the study by Amzali et al. [57], this may not be sufficient for FSHD patients. Thus, supplementation strategies, alongside a Mediterranean diet, could further enhance the nutritional management of FSHD, alleviating symptoms and potentially counteracting disease progression (Figure 1).

### 5.2. Proteins and Essential Amino Acids

While specific clinical guidelines for protein intake in FSHD patients are still lacking, the muscle atrophy observed in this disease share similarities with that seen in sarcopenia. Sarcopenic individuals, like FSHD patients, experience a reduction in muscle mass, strength, and function [58,59,60]. As a result, FSHD patients may benefit from a daily protein intake similar to the recommended for sarcopenic individuals [59,61,62]. In addition to the total daily protein intake, both the timing of protein consumption and the individual doses per meal play a pivotal role in optimizing MPS [60]. Specifically, in the context of sarcopenia, research suggests that a diet providing 1.2–1.5 g of proteins/kilogram of body weight/day, with protein doses of 25–30 g every 3–4 h, is ideal for stimulating MPS throughout the day [59,61,62,63]. Meeting daily protein requirements is particularly important in conditions characterized by progressive muscle atrophy, such as FSHD [2,32]. Inadequate protein intake may lead to a negative protein balance, which could further exacerbate the muscle atrophy observed in these conditions [60].

Furthermore, an inadequate daily intake of even a single essential amino acid (EAA) can impair MPS [64]. Beyond the importance of meeting the requirements for each EAA through dietary proteins, supplementation with free-form EAAs, regardless of exercise stimulus, has been shown to provide significant benefits [64]. Among EAAs, leucine is a key regulator of MPS via activation of the mammalian target of rapamycin kinase complex 1 (mTORC1) [40]. Ensuring an adequate supply of this EAA (1–3 g) in each meal is crucial for optimizing MPS [62,65], especially in hypercatabolic states [66], such as FSHD. Even regarding EAAs, both the timing and quantity are of great importance. Specifically, a dose of 10–12 g of EAAs, including 1–3 g of leucine, every 3–4 h throughout the day is considered optimal for maximizing MPS [63].

### 5.3. β-Hydroxy-β-Methylbutyrate

β-hydroxy-β-methylbutyrate (HMB) is an active metabolite of the amino acid leucine, known to exert beneficial effects on muscle mass [67]. Similar to leucine, HMB upregulates MPS via the mTORC1 pathway [67]. However, only approximately 5% of leucine is converted into HMB [67] and plasma HMB concentration appears to be positively associated with muscle mass and strength [68]. Future studies should investigate if FSHD patients, who experience progressive muscle mass and strength loss, may exhibit altered endogenous production of HMB and could therefore benefit from HMB supplementation. Several studies have addressed the effects of HMB supplementation, either alone or in combination with other amino acids, in various clinical settings in which muscle atrophy is characterized, including sarcopenia [69,70,71]. Overall, the results suggest that HMB supplementation may be a valuable nutritional supplement to counteract muscle mass atrophy. Available data suggest that the supplementation of HMB at doses of 1.5–3 g/day for at least one year is considered safe for humans [67].

### 5.4. Vitamin D

Muscle atrophy is regulated by a complex interplay of genetic, epigenetic, and behavioral mechanisms, with environmental and nutritional factors also playing a significant role. Among these factors, vitamin D, through its active form (1,25-dihydroxyvitamin D (1,25(OH)_2_D_3_)) and its nuclear vitamin D receptor (VDR), stands out as a key factor in regulating muscle function [72]. Notably, vitamin D deficiency has been associated with muscle pain, weakness, atrophy, and an increased risk of sarcopenia [73]. Potential underlying mechanisms include breakdown pathways, impaired mitochondrial function, oxidative damage, and increased adiposity [72]. While evidence is not unanimous, several studies suggest that vitamin D supplementation, either alone or in combination with other nutrients, such as amino acids or proteins, may be an effective strategy to enhance muscle health in both healthy individuals [74,75,76] and clinical populations [77,78,79]. However, to the best of our knowledge, data on the effects of vitamin D supplementation in the FSHD is lacking. Considering the above evidence, future studies should investigate whether vitamin D supplementation could provide benefits for patients with FSHD, where muscle atrophy is an intrinsic feature of the disease and may be further exacerbated by potential vitamin D deficiency.

### 5.5. Strategies for Proper Muscle Hydration

Cellular hydration status is an important factor controlling cellular protein turnover [80]. In fact, protein synthesis and protein degradation are influenced in opposite directions by cellular swelling (increase in cellular hydration) and shrinkage (decrease in cellular hydration), respectively [80]. In particular, cellular swelling acts as an anabolic proliferative signal, while cellular shrinkage is catabolic and antiproliferative. It is therefore plausible to hypothesize that the reduced ICW found in FSHD patients [12] may contribute to muscle protein catabolism. Therefore, achieving and maintaining proper hydration may help mitigate FSHD-related impaired muscle hydration. For this purpose, in addition to ensuring adequate hydration with water, preferably enriched with mineral salts, to meet individual needs, resistance exercise and specific supplementation strategies, such as creatine monohydrate, could offer additional benefits where applicable. Resistance training has been shown to promote increases in ICW, thus resulting in muscle hypertrophy [81,82,83]. Creatine, beyond its well-established role in energy metabolism [81], has also been shown to enhance ICW retention [84], which may further support cellular function and overall physical efficiency.

### 5.6. Supplements for Gut Microbiota Health

As previously discussed, the gut–muscle axis suggests that alterations in gut microbiota may lead to changes in muscle function, and vice versa [41,42]. To the best of our knowledge, the gut microbiota characteristics in FSHD patients have not yet been investigated. Since alterations in gut microbiota are observed in individuals with conditions associated with muscle atrophy, such as sarcopenia [85,86], it is plausible to hypothesize that it may also be altered in patients with FSHD, potentially contributing to the peculiar muscle impairments.

Pre-clinical and clinical studies utilizing probiotics have shown beneficial effects on protein synthesis and breakdown by modulating inflammation, nutrient metabolism, and oxidative stress [87,88]. Interestingly, such effects would provide a wide spectrum contrast for FSHD symptoms, since, as previously discussed, MPS, MPB, oxidative stress, and inflammation are impaired traits of the disease. This suggests the importance of future experiments in this cohort of patients.

## 6. Discussion

To the best of our knowledge, only five studies have investigated nutritional interventions in the FSHD population, involving a total of 131 patients [44,45,46,47,48]. The most consistent evidence comes from studies investigating the effects of antioxidant and anti-inflammatory supplements, which collectively involved 78 patients [44,45,46]. One study investigated protein and carbohydrate supplementation following aerobic training sessions in 41 patients [47], while another focused on creatine monohydrate supplementation in 12 patients [48]. While studies on antioxidant/anti-inflammatory [44,45,46] and protein–carbohydrate supplementations [47] proposed these interventions as potential strategies to enhance the antioxidant and anti-inflammatory response and contrast MPB, respectively, creatine monohydrate did not show significant improvements in muscle mass and function.

In contrast, data on specific dietary patterns are still lacking. Based on evidence from other populations that share pathological characteristics of FSHD (such as inflammation and oxidative stress), we have addressed these gaps by discussing promising nutritional approaches. Particularly, the Mediterranean diet, one of the dietary patterns with a wide range of health benefits [89], could also be a promising dietary model for FSHD. Thanks to its peculiar composition, this diet provides valuable nutrients with the potential to contrast inflammation and oxidative stress [52,53,54], which are typical of FSHD [12]. Furthermore, combining the Mediterranean diet with an antioxidant and anti-inflammatory supplementation strategy could offer an alternative to glucocorticoids, which are potent anti-inflammatory agents commonly used in FSHD [90,91,92]. This approach, if supported by future studies, may provide a long-term strategy, mitigating the adverse effects of glucocorticoids (such adrenal atrophy, osteoporosis, and immune suppression) which raise concerns about their prolonged use [92].

Furthermore, a Mediterranean diet enriched in proteins could help contrast the muscle atrophy typical of FSHD. Exercise is widely recognized as an effective strategy for enhancing muscle mass and function in both healthy individuals [93,94,95] and diseased cohorts [96,97], including FSHD [23,24,25,26,27]. However, as previously mentioned, FSHD patients often face limitations in their exercise capacity [9,10,11]. As a result, nutrition may offer an important alternative for maintaining and improving muscle mass and function [60]. Currently, there are no guidelines regarding the recommended daily protein intake for this patients’ cohort. Based on data from sarcopenic populations, which could lay the foundation for future studies in FSHD, we have addressed this gap. Nutritional interventions involving protein supplementation have demonstrated beneficial effects on muscle health, not only in healthy individuals [98] but also in clinical populations [61,62,99], including FSHD [47]. However, the evidence in FSHD [47] is based on a single study that assessed the short-term effects, highlighting the need for future research involving larger cohorts and longer follow-up periods. To better support MPS, we have discussed the potential of supplementation with HMB and vitamin D. Both have been associated with improvements in muscle atrophy and function in several sarcopenic cohorts [69,70,71,77,78,79] and may, therefore, offer additional benefits in FSHD as well.

Improvement in muscle function may also be supported by creatine monohydrate supplementation. Creatine monohydrate supplementation has been shown to increase muscle creatine and phosphocreatine concentrations, enhancing exercise capacity and training adaptations, which, in turn, improve muscle mass and strength [100]. In addition to its application in sports and training contexts, creatine monohydrate supplementation has been investigated in various clinical populations, including patients with muscular dystrophies. The most robust evidence is reported for patients with Duchenne muscular dystrophy, where creatine monohydrate supplementation has been associated with improvements in strength [101,102,103], perceived fatigue [101,104], and gait performance [105]. Although the study by Walter et al. [48] did not report significant benefits, it involved a small sample size and was of short duration. In light of these considerations and considering that creatine monohydrate supplementation is regarded to be safe across all age groups even in diseased populations [100], further investigation into its potential use in FSHD patients is warranted.

Finally, FSHD may represent a disease that could potentially benefit, at least in part, from the positive effects of probiotics supplementation. While no specific data are currently available regarding the gut microbiota in this cohort of patients, pre-clinical and clinical studies [87,88,106,107] from populations that share some of the pathological characteristic of FSHD offer promising insights into the potential role of probiotics.

In light of the results discussed in this review, only antioxidant and anti-inflammatory interventions demonstrated benefits in improving both the inflammatory and oxidative profiles, as well as muscle function, in the FSHD population. Although post-exercise protein–carbohydrate supplementation was effective in reducing MPB, it did not lead to additional functional improvements beyond those achieved through training. Additionally, creatine supplementation was not associated with significant improvements. The limitations of these two studies may be attributed to their relatively short duration and, in the case of the creatine supplementation, to the small sample size. Therefore, from a clinical standpoint, antioxidant and anti-inflammatory supplements could be integrated as evidence-based approaches in current disease management strategies. From a research perspective, protein and creatine supplementation warrant further investigation, involving larger cohorts and longer durations. Moreover, although still theoretical and not yet directly applicable to FSHD, a Mediterranean diet rich in protein, potentially enhanced by supplementation strategies including EAAs, HMB, vitamin D, and probiotics, could serve as a valuable complement to current FSHD management and lay the foundation for future studies to assess its long-term effects on the health and quality of life of FSHD patients.

## 7. Conclusions

In this review, we have addressed the critical aspects related to the nutritional management of patients with FSHD, aiming to highlight the current gaps in the literature and suggesting future research directions. Although specific nutritional guidelines have yet to be established, a dietary approach based on a Mediterranean-type diet, supplemented with specific nutrients and/or nutraceuticals, may offer a promising long-term management strategy for patients with FSHD. Existing data emphasize the potential therapeutic antioxidant and anti-inflammatory effects of several compounds, including flavonoids, curcumin, L-carnitine, coenzyme Q10, and omega-3, as well as vitamins C and E, and minerals such as zinc and selenium. However, evidence on the effects of proteins and creatine supplementation for improving muscle mass and strength remain limited. Furthermore, the potential benefits of EAAs, HMB, and vitamin D in contrasting muscle atrophy and enhancing muscle function are yet to be explored. Targeted nutritional interventions that address key pathological factors in FSHD, including reduced muscle mass, increased fat mass, inflammation, and oxidative stress, may provide valuable strategies for alleviating symptoms and slowing disease progression. When combined with tailored exercise programs, such interventions could form an effective long-term management approach for patients with FSHD. Future studies are required to define optimal treatment protocols, taking into consideration the multifaceted nature of the disease.

## Figures and Tables

**Table 1:** Available studies on nutritional interventions in the FSHD population.

Authors (year)	Sample	Nutritional Interventions (Duration)	Targeted Clinical Features	Main Outcomes
Passerieux et al. (2015) [44]	53 patients, of which 26 were in the supplemented group (15 males and 11 females, mean age of 42.4 ± 10.2 years) and 27 were in the placebo group (13 males and 14 females, mean age of 37.6 ± 9.3 years).	Vitamin C, E, zinc gluconate, and selenomethionine (17 weeks)	Inflammation and oxidative stress	↓ oxidative stress markers ↑ muscle function, muscle maximal voluntary contraction, and resistance
Sitzia et al. (2019) [45]	5 patients (5 males, mean age of 36.8 ± 9.8 years)	Flavonoids and omega-3 (24 weeks)	Inflammation and oxidative stress	↑ 6 min walk test and knee extension
Wilson et al. (2024) [46]	20 patients (mean age 39.1 ± 11.6 years)	Vitamin C, E, zinc gluconate, and selenomethionine (17 weeks)	Inflammation and oxidative stress	↓ muscle fat ↑ muscle mass and strength
Andersen et al. (2015) [47]	41 patients (21 males and 20 females, range age 19–65 years)	Post exercise protein–carbohydrate supplementation (12 weeks)	Muscle atrophy	↓ MPB
Walter et al. (2000) [48]	12 patients (mean age 37 ± 19 years)	Creatine monohydrate (8 weeks)	Muscle strength deficit	no significant improvements

FSHD, facioscapulohumeral dystrophy; MPB, muscle protein breakdown.

## Data Availability

No new data were created or analyzed in this study.
